# Expanding Bias-instability of MEMS Silicon Oscillating Accelerometer Utilizing AC Polarization and Self-Compensation

**DOI:** 10.3390/s20051455

**Published:** 2020-03-06

**Authors:** Yang Zhao, Guoming Xia, Qin Shi, Anping Qiu

**Affiliations:** School of Mechanical Engineering, Department of Instrument Science and Technology, Nanjing University of Science and Technology, Nanjing 210094, Jiangsu, China; zhaoyang0216@njust.edu.cn (Y.Z.); xiaguoming@njust.edu.cn (G.X.); sqinhy@njust.edu.cn (Q.S.)

**Keywords:** MEMS oscillating accelerometer, AC polarization, self-compensation, flicker noise, bias-instability, front-end TIA

## Abstract

This paper presents a MEMS (Micro-Electro-Mechanical System) Silicon Oscillating Accelerometer (SOA) with AC (alternating current) polarization to expand its bias-instability limited by the up-converted 1/f noise from front-end transimpedance amplifier (TIA). In contrast to the conventional DC (direct current) scheme, AC polarization breaks the trade-off between input transistor gate size and white noise floor of TIA, a relative low input loading capacitance can be implemented for low noise consideration. Besides, a self-compensation technique combining polarization source and reference in automatic-gain-control (AGC) is put forward. It cancels the 1/f noise and drift introduced by the polarization source itself, which applies to both DC and AC polarization cases. The experimental result indicates the proposed AC polarization and self-compensation strategy expand the bias-instability of studied SOA from 2.58 μg to 0.51 μg with a full scale of ± 30 g, a 155.6 dB dynamic range is realized in this work.

## 1. Introduction

Nowadays, MEMS accelerometers are ubiquitous in automobile, consumer electronics and guidance/inertial navigation systems [1,2,3,4,5]. The most available MEMS accelerometers are capacitive and based on a displacement sensing mechanism. Although some capacitive accelerometers have realized μg-grade performance, it suffers from poor linearity and a harsh tradeoff between sensitivity and bandwidth, hence a limited dynamic range [6,7]. On the other hand, MEMS silicon oscillating accelerometer, which based on force sensing, is a promising counterpart. Sub 0.1-μg grade accuracy has been reported with sufficient bandwidth (up to several kilohertz) and dynamic range (> 140dB) [8,9]. 

MEMS SOA is essentially two resonators connected to a proof mass. Applied acceleration on the proof mass generates tensile/compressive forces on the two resonators, respectively, changing their resonant frequencies. Since the acceleration is frequency modulated, its sensitivity is no longer restricted by the natural frequency of the proof mass, moreover, ultra-high-Q mode (Q > 100,000) can be adopted in MEMS resonators to realize low mechanical noise and better immunity to parametric noise from sustaining circuit without the penalty of bandwidth [10]. In addition, a scale factor with ~10 ppm grade nonlinearity and ~1 ppm stability could be achieved over a large input acceleration range (± 20 g) without any additional feedback control in contrast to capacitive counterpart [11]. Besides, the quasi-digital frequency output dynamic range does not rely on the supply voltage and can be readily demodulated by a counting technique [12], thus preventing the usage of a costly ADC, and a readout chip with μW grade power is realizable [13,14].

Bias-instability derived from Allan variance is a metric of the lowest stability that a SOA can achieve when an average process is adopted [15], which is the most important performance for navigation systems where the near DC error is at top priority [16]. Bias-instability is decided by both flicker noise floor and temperature drift. Numerous researches focused on the reduction of temperature drift by means of proper mechanical sensing element and package design as well as post compensation techniques [17,18,19]. On the other hand, only a few articles focused on flicker noise minimization. [20] firstly pointed out the 1/f noise in the automatic gain control (AGC) loop can be up-converted to oscillation amplitude and show up in the frequency domain through the amplitude-stiffness (A-S) nonlinear effect. Chopper stabilization and a gain-boosted gm cell can be utilized to get rid of such up-converted 1/f noise from AGC [21,22]. Except for the few articles about 1/f noise in AGC, the bias-instability contributed from other sources is unexplored.

To acquire sufficient motional resistance, the resonant beam of a MEMS resonator is usually polarized to a DC level several times higher than the supply rail, which is generated by a DC charge pump. This polarization voltage contains a large 1/f noise component and could be also up-converted to oscillation amplitude. Actually, this assumption has been proved in CMOS-MEMS oscillators—as reported in [23], a clear 1/f^3^ phase noise slope from polarization is observed. 

It has been reported that the MEMS resonator can also be polarized by an AC source, which reduces the frequency drift introduced by charges on the associated capacitive electrodes [24,25]. Recently, such AC polarization technique is adopted in MEMS SOA to down-convert a 157.5 kHz oscillation frequency to 10 kHz; in this way, the bandwidth and noise requirement of front-end TIA is greatly reduced [26]. Nevertheless, the impact of AC polarization on 1/f noise and bias-instability of SOA is not yet well-explained. 

In this work, we point out the 1/f noise in TIA common mode voltage is more troublesome than the polarization voltage on resonant beam. An AC polarization strategy is proposed to remove the up-converted 1/f noise from TIA and a self-compensation method is put forward to cancel the 1/f noise and drift from the AC polarization source. The proposed techniques improve the bias-instability of SOA from 2.58 μg to 0.51 μg.

The rest of paper is organized as follows. Section 2 briefs the fundamentals of MEMS SOA and its readout circuit adopted in this work. Section 3 explains why AC polarization can deal with the 1/f noise from front-end TIA and the closed-loop spectrum measurement results are presented. Section 4 introduces the AC polarization source self-compensation idea as well as its implementation with chopper-stabilized AGC. The experimental results of MEMS SOA with AC polarization are presented in Section 5, and Section 6 concludes the paper.

## 2. MEMS SOA Overview

**Figure 1:**
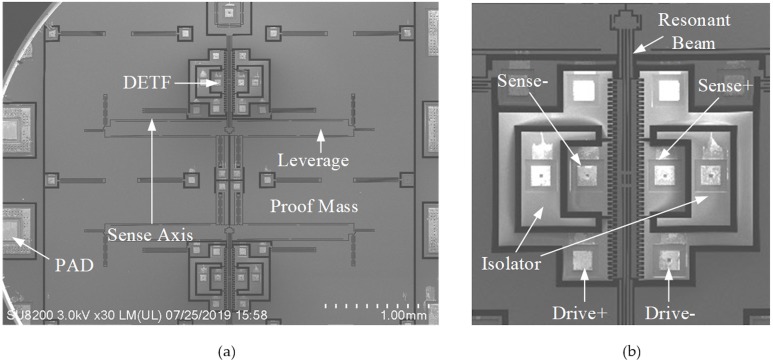
Scanning electron micrographs of the (**a**) MEMS SOA adopted in this work and (**b**) a zoom in view of its DETF.

The scanning electron micrograph of the MEMS SOA adopted in this work is presented in Figure 1a. It consists of a proof-mass attached to two double-ended tuning fork (DETF) resonators via a micro-leverage as force amplifier. When the proof mass is subject to external acceleration along sense axis, the inertial force will load the two DETFs differentially and result in frequency shifts of their natural frequencies. The frequency difference of the two DETFs is therefore a measure of the applied acceleration. 

A zoom-in view of the bottom DETF is given in Figure 1b, which includes four sets of combs for differential excitation and readout, a resonant beam and two sets of isolation electrodes to minimize feedthrough between drive and sense combs. The nominal frequency of each DEFT is 18 kHz, with an acceleration sensitivity of 70 Hz/g with the assistance of micro-leverage. The target full scale is ± 30 g.

The sensing element die is fabricated in an 80-μm SOI (Silicon-On-Insulator) process with a high aspect ratio up to 1:30 [27]. The die is realized with three wafers: the substrate, the device layer and the cover. The device layer is 80 μm-thick and manufactured using Deep Reactive Ion Etching on a SOI wafer. The cover and the active SOI layer are joined by Au/Si eutectic bonding, forming a hermetic cavity to maintain the vacuum for the resonator. 

The typical Q-factor of fabricated MEMS resonators is around 200,000, which guarantees a low phase noise level and a realizable low drive voltage under low supply voltage. Since the acceleration is frequency modulated, the SOA bandwidth is not restricted by the resonator’s high Q-factor. The theoretical bandwidth of this SOA is beyond 1 kHz, limited by the resonance frequency of the proof mass. 

**Figure 2:**
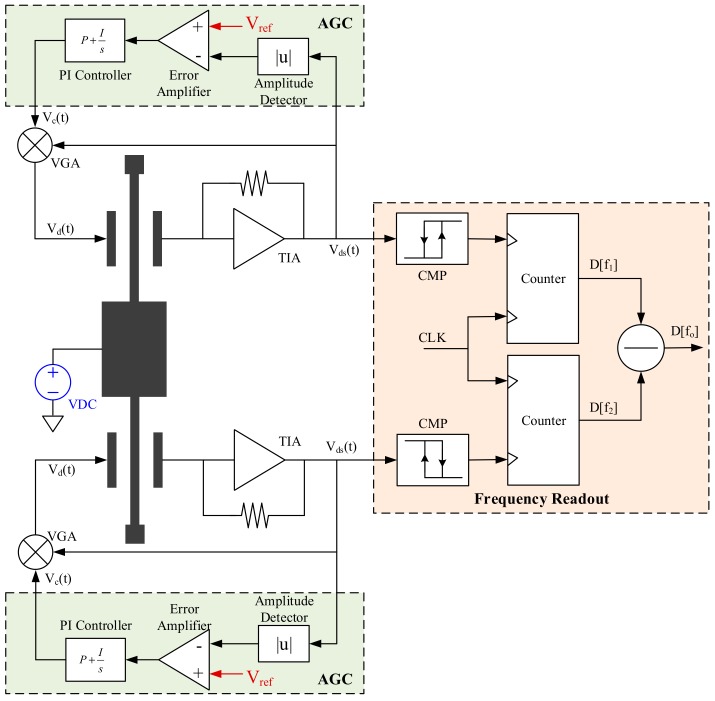
Block diagrams of a MEMS SOA oscillation loop and frequency readout system.

To track their resonant frequencies, the two DETFs are embedded into two identical oscillation loops, and Figure 2 provides a representative one. Normally, a relative high DC polarization voltage, V_DC_, is applied on the proof mass to provide adequate gain for electrostatic actuation and detection. The front-end TIA picks up the resonator’s motional current and converts it to voltage, V_ds_(t), which is in-phase with the resonator’s oscillation velocity. The AGC extracts the instantaneous oscillation amplitude and compares it with a preset reference, V_ref_, to generate an error control signal, V_c_(t). The feedback drive voltage is regulated by V_c_(t) via a variable gain amplifier (VGA) to sustain an oscillation with a stable amplitude. A comparator reshapes the output of TIA and a counter digitalizes the oscillation frequency. The acceleration output is realized via a subtraction of the two output frequencies.

## 3. AC Polarization Technique

### 3.1. Why AC Polarization?

Bias-instability, derived from Allan Variance, is one of the most important metrics of MEMS SOA, which sets its ultimate precision. Apart from thermal drift, bias-instability is limited by the 1/f floor in SOA electromechanical system. Since 1/f noise only shows up in low frequency range, usually below 1 kHz, it does not seem to disturb the oscillation signal at ω_0_. However, the 1/f noise in oscillation amplitude could convert to oscillation frequency due to the amplitude-stiffness (A-S) nonlinear effect [28]. As studied in [21,22], the 1/f noise in AGC could up-convert to oscillation amplitude, chopper stabilization and gain-boosted error amplifier could solve this trouble. In this work, we focus on the 1/f noise introduced by the front-end TIA and polarization voltage, V_DC_.

In SOA oscillation loop with AGC, the oscillation amplitude, V_ds_, is a replica of V_ref_ in AGC with a scale factor of α. The mechanical oscillation displacement can be derived as

(1) $$x=\frac{\alpha V_{ref}}{\omega_{0}V_{DC}R_{ds}\frac{C_{ds0}}{L_{d0}}}$$

in which V_DC_ is the polarization voltage applied across the sense comb, R_ds_ represents the transimpedance of TIA, C_d0_ and L_d0_ are the static capacitance and overlap of sense comb. Apparently, the 1/f noise in V_DC_ disturbs the oscillation displacement and shows up in SOA frequency output via A-S effect.

**Figure 3:**
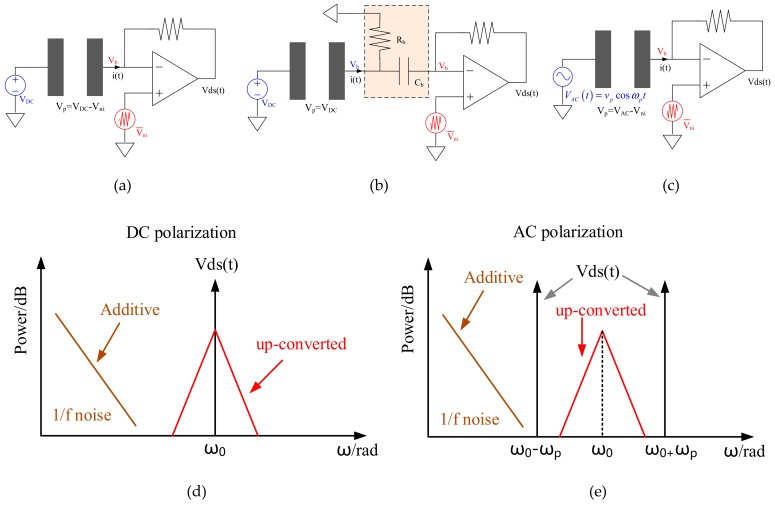
Illustrations of MEMS sense comb with three polarization modes (**a**) DC polarization; (**b**) DC polarization with R-C passive filter; (**c**) AC polarization and TIA output spectrum with; (**d**) DC and (**e**) AC polarization.

As shown in Figure 3, the polarization voltage across the sense comb depends on both V_DC_ and V_b_. The noise from V_DC_ is neglected temporarily, since a relative low noise external reference can be adopted, but it is not practical for V_b_. Figure 3a,b present two commonly used ways to apply V_b_ on the sense electrode. In Figure 3a, V_b_ is supplied by the common mode voltage of front-end TIA (V_b_ is assumed to be 0V hereinafter for simplification). V_ni_ models the input referred voltage noise of TIA, and its 1/f noise part can be expressed as

(2) $${\bar{v}}_{ni}^{2}=\frac{K}{WL}\frac{1}{f}$$

where K is a process related constant, W and L are the width and length of the input transistor in TIA. The only way to reduce 1/f noise in V_b_ is to increase the size of the input transistor, but it increases the gate capacitance as well. As explained in [27], the stability, gain-bandwidth product (GBP) and noise floor of TIA highly depend on its input loading capacitance, the 1/f noise of V_b_ must be compromised and became the dominant contributor of bias-instability other than AGC.

Figure 3b presents an alternative solution: a R-C passive filter is inserted between sense electrode and front-end TIA, which allows the motional current flow through C_b_ and apply DC bias via R_b_ [29,30]. In this case, the polarization voltage across the sense comb is free from the 1/f noise in TIA, but the cut-off frequency of this R-C network shall be low enough and hard to be integrated on-chip (i.e., C_b_ > 1nF, R_b_ > 10MΩ). Besides, these off-chip components exclude the possibility of chip-to-chip bonding and worsen the SOA performance due to the added parasitics.

To break the harsh tradeoff between 1/f noise in V_b_ and TIA input loading capacitance, AC polarization could be a superior candidate. As proposed in Figure 3c, a sinusoid waveform is applied on the resonant beam and the polarization voltage across the sense comb is then |V_AC_(t)-V_ni_|. In this case, the oscillation displacement will be modulated by V_AC_(t) to generate motional current, but the 1/f noise of V_b_ still up-converted to ω_0_, as illustrated in Figure 3d,e. Consequently, AC polarization provides a solution to further improve the bias-instability of MEMS SOA.

The detailed derivation of how the MEMS resonator works under AC polarization will be introduced in Section 3.2. It is important to note that the 1/f noise in DC and AC polarization source up-converts to oscillation amplitude in the same way. The self-compensation method in Section 4 will handle this issue.

### 3.2. Fundamentals of AC Polarization

**Figure 4:**
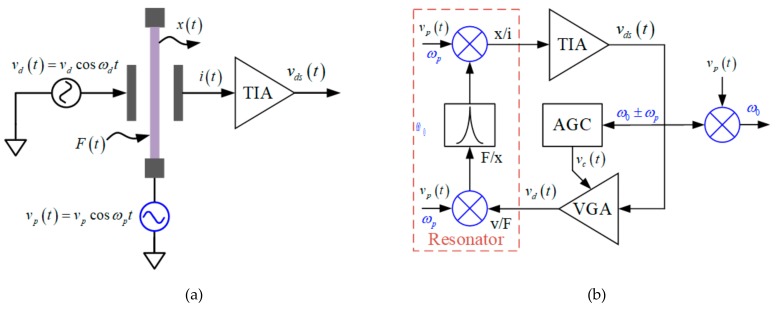
Schematics of MEMS resonator adopting AC polarization in (**a**) open-loop driving and (**b**) closed oscillation modes.

The concept of AC polarization can be illustrated in Figure 4a. A drive voltage, V_d_(t), is applied on the force electrode and an AC voltage, V_p_(t), polarizes the resonant beam. A TIA picks up the motional current generated from the detection comb. 

Assuming both the drive voltage and polarization voltage are sinusoids, as shown in Figure 4. The drive force can be derived as below

(3) $$\begin{array}{l} F\left(t\right)=\frac{1}{2}\frac{C_{d0}}{L_{d0}}{\left(v_{d}\cos\omega_{d}t-v_{p}\cos\omega_{p}t\right)}^{2}=\alpha v_{d}^{2}\cos2\omega_{d}t+\alpha v_{p}^{2}\cos2\omega_{p}t\\ \;\;\;\;-\alpha v_{d}v_{p}\left[\cos\left(\left(\omega_{d}+\omega_{p}\right)t\right)+\cos\left(\left(\omega_{d}-\omega_{p}\right)t\right)\right] \end{array}$$

where

(4) $$\alpha =\frac{C_{d0}}{2L_{d0}}$$

Since a high-Q resonator filters out-off-band signals greatly, the resonant beam is mandatory to oscillate at is intrinsic frequency, ω_0_. It indicates only when the applied drive voltage frequency ω_d_ *=* ω_0_ ± ω_p_ or ω_d_ *=* ω_0_/2, can it drive the resonator effectively. The gain of the first term in (3) is v_p_/v_d_ times lower than the third term, as a result, the oscillation can only take place at ω_d_ *=* ω_0_ ± ω_p_. Accordingly, the drive force can be simplified as

(5) $$F\left(t\right)=-\alpha v_{d}v_{p}\cos\omega_{0}t$$

At resonance, the displacement is amplified by *Q*/*k* and has a π/2 lag behind, which can be expressed as

(6) $$x\left(t\right)=-\alpha v_{d}v_{p}\frac{Q}{k}\cos\left(\omega_{0}t-\frac{\pi }{2}\right)$$

Owing to the movement of resonant beam, the detection capacitance is also time-variable as given in (7)

(7) $$C_{ds}\left(t\right)=C_{ds0}\left(1-\frac{x\left(t\right)}{L_{d0}}\right)$$

The motional current consequently comes from two sources, namely, variable capacitance and variable polarization voltage.

(8) $$i\left(t\right)=\frac{dQ}{dt}=v_{p}\left(t\right)\frac{dC\left(t\right)}{dt}+C\left(t\right)\frac{dv_{p}\left(t\right)}{dt}$$

(9) $$i\left(t\right)=-\left(\omega_{0}+\omega_{p}\right)\alpha^{2}v_{p}^{2}v_{d}\frac{Q}{k}\sin\left(\omega_{0}+\omega_{p}\right)t-\left(\omega_{0}-\omega_{p}\right)\alpha^{2}v_{p}^{2}v_{d}\frac{Q}{k}\sin\left(\omega_{0}-\omega_{p}\right)t-\omega_{p}C_{ds0}v_{p}\sin\omega_{p}t$$

The first and second terms in (9) are both resonant current in the AC polarization approach; their corresponding motional resistance can be derived as below [31].

(10) $$R_{1}=\frac{v_{d}}{i_{\omega_{0}+\omega_{p}}}=\frac{\omega_{0}}{\left(\omega_{0}+\omega_{p}\right)}\frac{k}{\alpha^{2}v_{p}^{2}\omega_{0}Q}\;R_{2}=\frac{v_{d}}{i_{\omega_{0}-\omega_{p}}}=\frac{\omega_{0}}{\left(\omega_{0}-\omega_{p}\right)}\frac{k}{\alpha^{2}v_{p}^{2}\omega_{0}Q}$$

In the DC polarization scheme, the resonator’s motional resistance is given by (11).

(11) $$R_{DC}=\frac{k}{4v_{p}^{2}\alpha^{2}\omega_{0}Q}$$

Apparently, the motional resistances R_1_ and R_2_ are 4x larger than R_DC_. In a closed oscillation loop, as presented in Figure 4b, the motional currents at ω_0_ ± ω_p_ are both fed back to generate drive voltage after the amplification of TIA, the total motional resistance, R_AC_, in AC polarization shall be R_1_||R_2_, which equals to 2 R_DC_. The additional 1/2 conversion gain from drive voltage to drive force in (3) shall be blamed for the 2x gain loss in AC polarization approach. It suggests the AC and DC polarization voltages shall be equal in rms value to maintain same motional resistance.

A current component at ω_p_ can also be found in (9), which is a pure feedthrough signal independent of the resonator’s motion. Although it can be filtered out when it fed back to the high-Q resonator, it is important to make sure the feedthrough will not saturate the TIA. For a resonator with differential detection capacitances, the feedthrough would be a common-mode interference and can be rejected by the TIA front-end. Nevertheless, mismatch of the detection capacitance or the front-end transimpedance always exists, the front-end gain shall be carefully chosen. 

**Figure 5:**
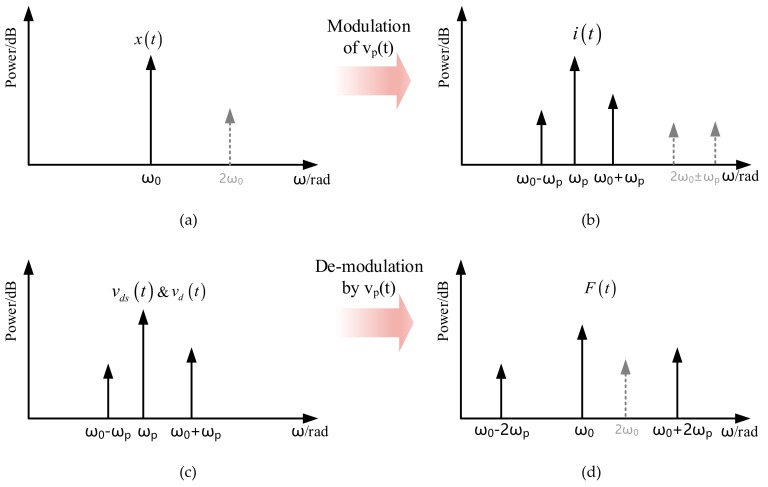
Frequency spectrums of (**a**) oscillation displacement (**b**) motional current (**c**) detection/drive voltage and (**d**) drive force in a MEMS resonator adopting AC polarization. Differential detection topology is assumed to cancel the feedthrough at ω_p_ after the amplification of TIA.

Figure 5 summarizes the frequency spectrums of four selected signals in a closed oscillation loop adopting an AC polarization approach. In brief, the oscillation displacement x(t) is modulated by v_p_(t) during the motional current conversion process. The amplified drive voltage is then demodulated by v_p_(t) when it fed back on the drive electrode to generate driving force.

Since the oscillator output is modulated to ω_0_ ± ω_p_, a demodulation process is necessary to recover the mechanical resonant frequency, ω_0_, as presented in Figure 4b.

In the closed oscillation loop, the drive voltage, v_d_(t), contains two components, ω_0_ ± ω_p_. Due to the square relationship between drive voltage and drive force, as given in (3), a drive force at 2ω_0_ will emerge and result in a corresponding displacement variation in (6). After the modulation of AC polarization, motional current at 2ω_0_ ± ω_p_ will show up. Since pure sinusoid drive voltage is supposed for simplification in the derivation procedure of (3)–(9), the 2nd harmonic related components are not included in (9), but drawn in Figure 5 in a dotted line.

### 3.3. Closed-Loop Oscillator Measurement

To verify the feasibility of AC polarization to sustain a stable oscillation, the startup period responses of MEMS SOA utilizing DC and AC polarization are measured. To make a fair comparison, the rms value of AC polarization is chosen to be equal to DC polarization value, 7V. The AC polarization frequency is set to be 13 kHz to reject the additive 1/f noise in V_b_ and guarantee the modulated sidebands both lie in the bandwidth of TIA.

**Figure 6:**
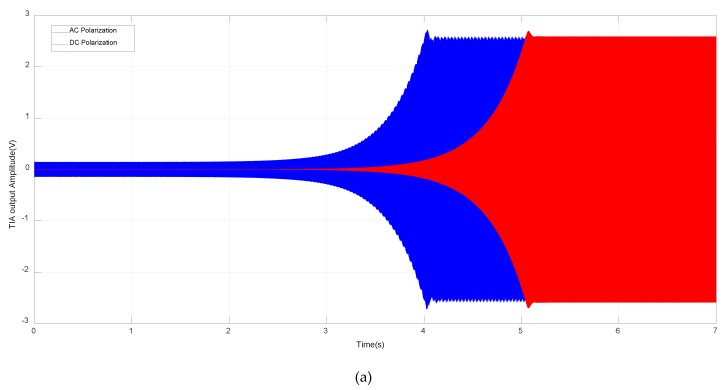

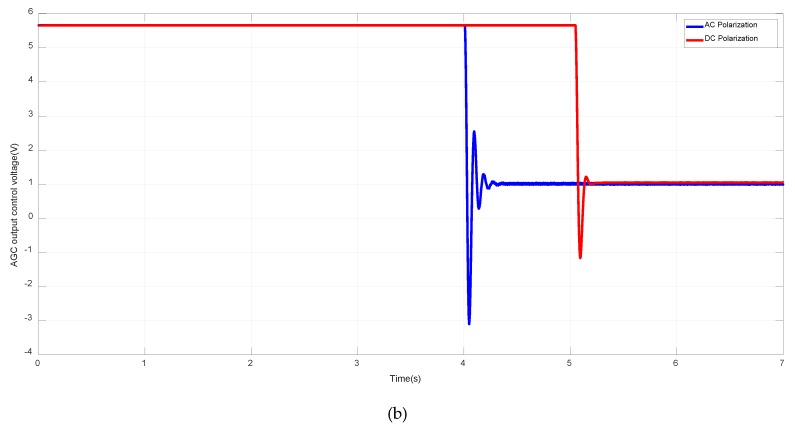
Startup process of (**a**) TIA output voltage, VDS(t) and (**b**) AGC output control voltage, Vc(t) under DC and AC polarization.

Figure 6 gives the startup response of TIA output, V_DS_(t), and AGC output, V_c_(t), under DC and AC polarization cases. The TIA output stable amplitudes are both 2.6 V, a preset value defined by AGC. The AGC output control voltages both converge to around 1V, which affirms the loop gains under DC and AC polarization are identical. Another important observation is that the startup time of AC polarization is about 20% shorter than its DC counterpart. The reason is not yet well-understood.

**Figure 7:**
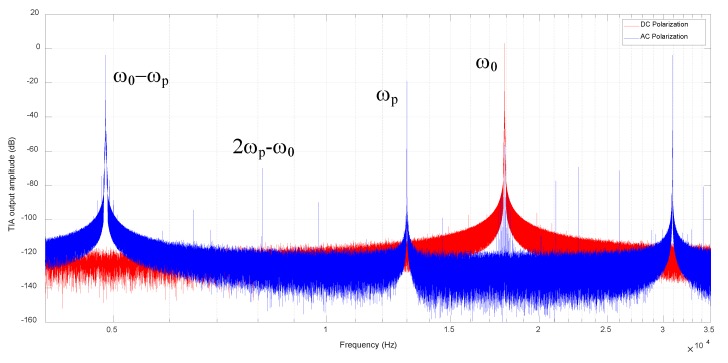
Frequency spectrums of TIA output voltage, VDS(t) under DC and AC polarization.

The comparison of TIA output spectrum is presented in Figure 7. The natural frequency of the MEMS resonator is 17.87 kHz; as expected in (9), tones of ω_0_ ± ω_p_ and feedthrough at ω_p_ are observed when AC polarization is applied. Moreover, feedthrough at ω_p_ and tones caused by 2nd harmonic of resonator and AC polarization can also be found in the spectrum such as 2ω_p_ - ω_0_ and 2ω_0_ - ω_p_. The feedthrough can be cancelled by injecting a compensation current at the input node of TIA, as explained in [26]. In this work, the feedthrough is still within the linear range of TIA and no cancellation is carried.

As discussed in 3.2, a closed oscillation loop contains 2ω_0_ related harmonic components. Limited to the window range, only peak at 2ω_0_ - ω_p_ is observed in Figure 7. The peak at 2ω_p_ - ω_0_ is also observed due to the distortion of applied AC polarization source. Taking a 2ω_p_ distortion of v_p_(t) into consideration in eq.8-9, motional current at 2ω_p_ ± ω_0_ will show up. Since the peak at 2ω_p_ + ω_0_ is beyond the window of Figure 7, only peak at 2ω_p_ - ω_0_ is observed.

Since the distortion of AC source is limited and the high-Q resonator filters out the 2ω_0_ drive force greatly, these 2nd harmonic related tones are at least 60 dB lower than the main peaks, which can be neglected.

## 4. Self-Compensation of AC Polarization Source

After AC polarization is adopted, the up-converted 1/f noise from front-end TIA is removed. However, the 1/f noise lies in the AC polarization source, V_p_, and amplitude control reference in AGC, V_ref_, still upconvert to the oscillation amplitude directly as given in (1).

**Figure 8:**
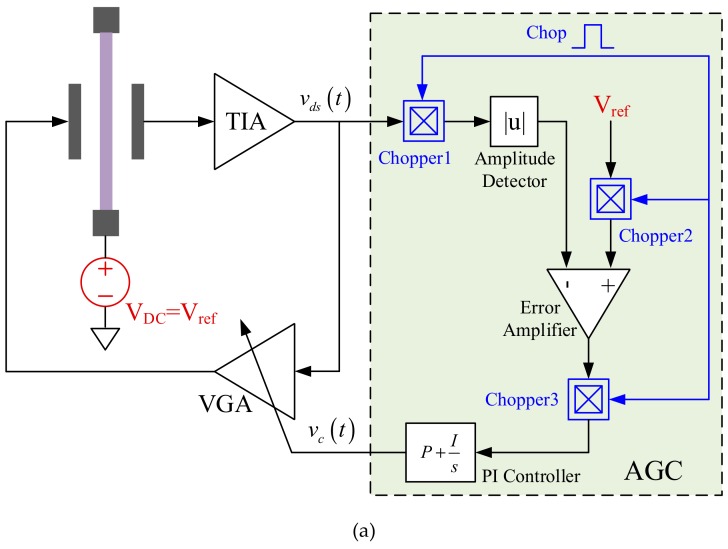

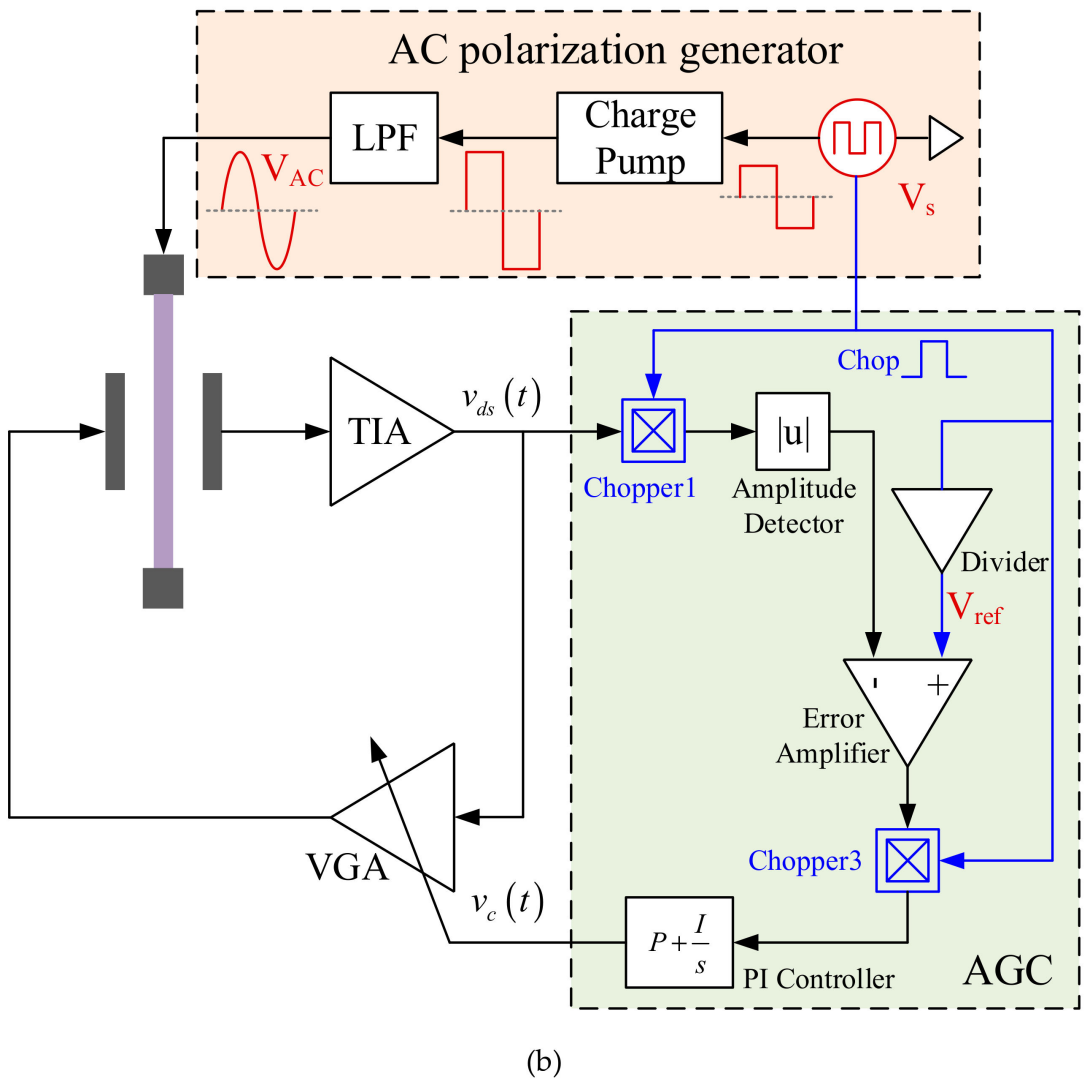
Schematics of closed-loop oscillator with polarization voltage self-compensation under (**a**) DC and (**b**) AC modes.

To further improve the 1/f noise limited bias-instability, a self-compensation method is proposed in this work. As (1) indicated, the oscillation displacement is a scale of V_ref_ to V_p_, which means the correlative variation of them can be cancelled automatically. From this point, a self-compensation strategy is proposed to correlate the two reference sources and Figure 8 gives its basic schematics under DC and AC polarization cases.

In DC polarization mode, such a self-compensation can be easily realized by sharing one voltage reference for V_DC_ and V_ref_, as shown in Figure 8a. Nevertheless, similar self-compensation under AC polarization mode is a little difficult, the reference in AGC must be AC now. Chopper-stabilized AGC introduced in [22] happens to be compatible with the intended self-compensation strategy. 

As shown in Figure 8b, a square wave provides references for both V_ref_ and V_AC_. The chopper 2 in conventional AGC is removed, and a divided V_s_ supplies as V_ref_ straight forward. The control clock of chopper1 and 3 is Vs as well to make it synchronize with V_ref_. For V_AC_, a charge pump is firstly utilized to boost Vs to a high level and a passive low-pass-filter depresses the high order harmonic components before applying it on the resonant beam. In such a way, the V_ref_ and V_AC_ are correlated as they did in DC polarization mode.

To verify the effectiveness of proposed self-compensation strategy, the AC polarization is firstly cut off from the proposed generator and connected with an independent AC source. The V_s_ in the proposed generator is manually altered and the MEMS resonator’s corresponding frequency changes are measured. After that, the proposed generator is enabled.

**Figure 9:**
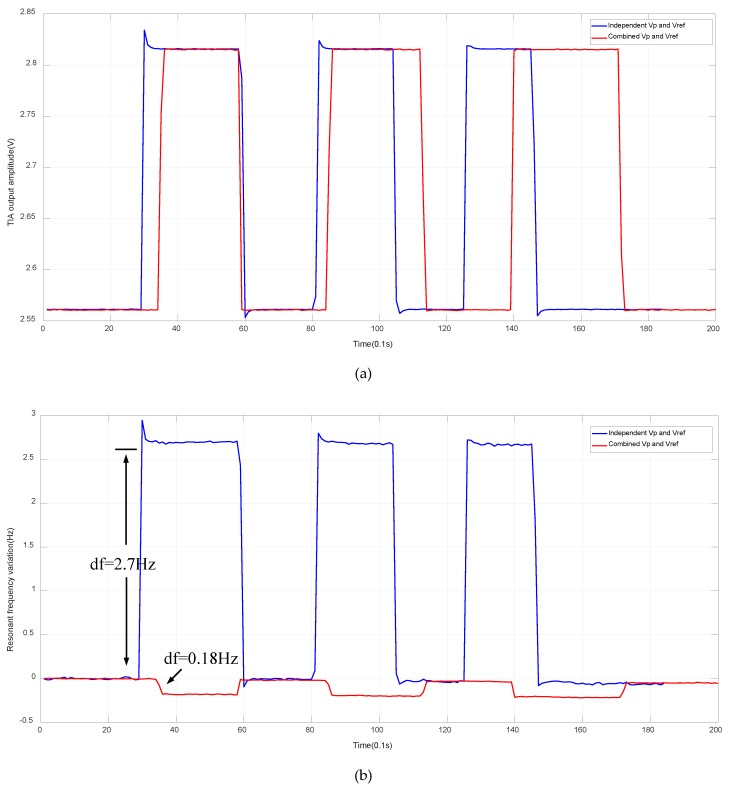
Variation comparison of (**a**) TIA output amplitude and (**b**) its frequency with self-compensation strategy or not.

As concluded from Figure 9a in both cases, the oscillation output voltages, V_DS_(t), are altered 10%. The self-compensation strategy reduces the oscillation frequency change from 2.7 Hz to 0.18 Hz, a 15x improvement, as presented in Figure 9b. The residual frequency variation is believed to be caused by the nonlinear behavior of the amplitude detector in AGC [32], which introduces slight mismatch between V_ref_ and amplitude of V_DS_(t).

## 5. SOA Experiment Results

### 5.1. Acceleration Response

Section 3 has verified the AC polarization is workable for a closed-loop oscillator, but its influences on the performance of MEMS SOA needed to be explored. A board-level readout circuit was adopted for quick verification. 

**Figure 10:**
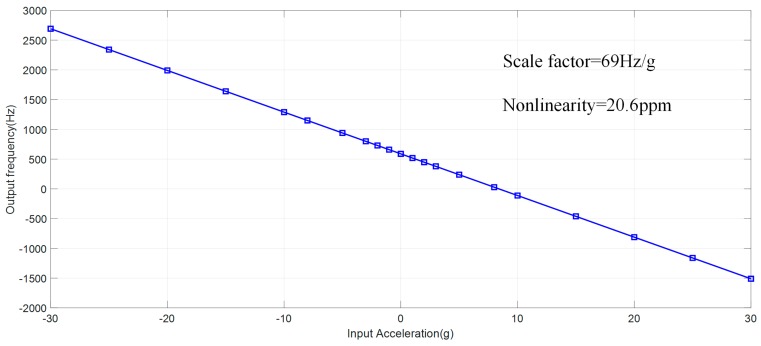
Acceleration response of SOA within a full scale of ± 30 g.

The acceleration response of the proposed SOA with AC polarization is tested firstly. The SOA is mounted on a centrifuge and ± 30 g input is applied. The measured response of proposed SOA is plotted in Figure 10, demonstrating a scale factor of 69 Hz/g with a maximum nonlinearity of 20.6 ppm.

### 5.2. Allan Variance

**Figure 11:**
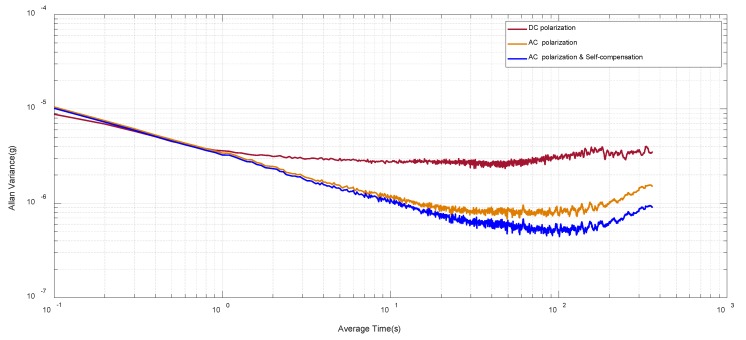
Allan variance test result under three polarization conditions, DC polarization, AC polarization and AC polarization with self-compensation.

The bias-instability of SOA with proposed AC polarization and self-compensation strategy is measured subsequently under 0 g input according to the IEEE std [33]. The SOA output is sampled for 1 h under three conditions, namely, (1) under DC polarization; (2) under AC polarization; and (3) AC polarization together with self-compensation. Their corresponding Allan Variance plots are presented in Figure 11.

The proposed AC polarization strategy advances the bias-instability from 2.58 μg to 0.83 μg, and the self-compensation further expands the bias-instability to 0.51 μg. A 5x improvement is realized in total, which benefits from the exclusion of 1/f noise and drift from front-end TIA and polarization source. Considering the ± 30 g full scale into consideration, the SOA with proposed AC polarization achieves a dynamic range of 155.6 dB.

Compared with our previous work in [27], a considerable bias-instability (0.6 μg to 0.51 μg) is achieved. However, a R-C bias tree is employed in [27] to filter out the 1/f noise from TIA. After the preliminary verification of AC polarization idea in MEMS SOA, a CMOS chip-level readout circuit will be developed subsequently to further improve the sensor accuracy.

## 6. Conclusions

This paper presents an AC polarization strategy for MEMS SOA to exclude the up-converted 1/f noise from front-end TIA to expand its bias-instability. Meanwhile, a relative low gate size of input transistors in TIA could be adopted to minimize the input capacitance loading, which improves the white noise dominated resolution as well. Besides, a self-compensation method to further depress the 1/f noise and drift from polarization source is proposed, which applies to both DC and AC polarization cases. The experimental result indicates the AC polarization and self-compensation strategy expand the bias-instability of SOA from 2.58 μg to 0.51 μg with a full scale of ± 30 g, a 155.6 dB dynamic range is realized in this work.
